# Efficiency of cell-based assays in detecting AChR antibodies in myasthenia gravis sera with low antibody concentrations as determined by radioimmunoprecipitation assay

**DOI:** 10.3389/fimmu.2025.1459423

**Published:** 2025-05-28

**Authors:** John S. Tzartos, Aigli G. Vakrakou, Katerina Karagiorgou, Vasiliki Zouvelou, Elisabeth Chroni, Valentina Damato, Francesca Beretta, Stavroula Salakou, Eirini Sfyroera, Dimitra Veltsista, Konstantinos Voumvourakis, Sotirios Giannopoulos, Georgios Tsivgoulis, Socrates Tzartos

**Affiliations:** ^1^ Second Department of Neurology ‘‘Attikon’’ University Hospital, School of Medicine, National and Kapodistrian University of Athens (NKUA), Athens, Greece; ^2^ First Department of Neurology, School of Medicine, Aeginition Hospital, National and Kapodistrian University of Athens, Athens, Greece; ^3^ Tzartos NeuroDiagnostics, Athens, Greece; ^4^ Department of Neurology, School of Medicine, University of Patras, Rio-Patras, Greece; ^5^ Department of Neurosciences, Drugs and Child Health, University of Florence, Florence, Italy; ^6^ Hellenic Pasteur Institute, Athens, Greece; ^7^ Department of Pharmacy, University of Patras, Patras, Greece

**Keywords:** myasthenia gravis, radioimmunoprecipitation assay, cell-based assay, AChR antibodies, immunology

## Abstract

**Objectives:**

We investigated whether the acetylcholine receptor (AChR) cluster cell-based assay (CBA) is effective in detecting AChR antibodies in sera from myasthenia gravis (MG) patients with low antibody concentrations, as determined by radioimmunoprecipitation assay (RIPA).

**Methods:**

In this retrospective diagnostic cohort study, 193 RIPA-positive sera from MG patients were analyzed. Following initial assessment using the gold-standard RIPA, samples were tested with a commercially available fixed CBA (F-CBA) and an in-house live CBA (L-CBA) to detect clustered AChR antibodies. Patients were classified into three groups based on RIPA levels to evaluate the sensitivity of each CBA. A subset of the cohort was blindly retested in a second laboratory to confirm results.

**Results:**

The sensitivity of L-CBA and F-CBA in detecting 36 sera with low AChR-antibody levels (1.0–2.8 nM) was relatively high for L-CBA (83.33%, 95% CI: 71.16%, 95.51%) and low for F-CBA (45.71%, 95% CI: 29.21% to 62.22%). Both CBAs were 100% sensitive for sera with AChR-RIPA values > 3 nM. Antibodies of RIPA+/CBA− sera could be immunoadsorbed on AChR-transfected cells equally well as those from RIPA+/CBA+ sera, indicating that CBA negativity was due to low antibody concentrations.

**Discussion:**

Overall, while AChR L-CBA demonstrates good sensitivity for detecting low concentrations of AChR antibodies, F-CBA performs less reliably in such cases. Since clustered AChR-CBAs can also identify antibodies that are not detectable by RIPA, we recommend that both RIPA and CBA be used together in the routine diagnosis of MG whenever possible. When available, L-CBA should be preferred over F-CBA due to its higher sensitivity.

## 1 Introduction

The radioimmunoprecipitation assay (RIPA) is the gold standard method for the identification of acetylcholine receptor (AChR) antibodies in myasthenia gravis (MG) (1, 2). Approximately 80%–85% of generalized and 50%–65% of ocular MG patients are anti-AChR-RIPA positive (3, 4). However, a major drawback of RIPA is its reliance on radioactive reagents. Additionally, 5%–10% of AChR-RIPA-negative MG patients have antibodies against Muscle-Specific Kinase (MuSK) or Low-Density Lipoprotein Receptor-Related Protein 4 (LRP4), yet some MG patients remain seronegative (SNMG) (5). Leite et al. (6) reported that a live cell-based assay (L-CBA) for the detection of AChR antibodies, using rapsyn to densely cluster AChRs on the cell membrane, could detect AChR antibodies in 66% of an AChR-RIPA-seronegative MG cohort. L-CBA aimed to replicate the high AChR density at the neuromuscular junction and facilitate the detection of low-affinity AChR antibodies that are strictly specific for the native AChR conformation. This finding was later confirmed in varying percentages (4%–50%) of SNMG patients (7–10) and was often found to be superior to RIPA (11–14).

Notably, recent studies using flow cytometry-based clustered AChR-CBA have shown that a significant proportion of AChR-RIPA-negative patients, ranging from 18.2% to 21%, tested positive for AChR antibodies (10, 13).

Nevertheless, the comparison between AChR-CBA and AChR-RIPA in MG sera with low-concentration RIPA AChR antibodies has not been sufficiently studied. Therefore, this investigation aimed to evaluate the performance of fixed CBA (F-CBA) and L-CBA in detecting low concentrations of AChR antibodies, as determined by RIPA, in a multicenter cohort of MG patients.

## 2 Materials and methods

### 2.1 Standard protocol approvals, registrations, and patient consent

This study was performed in accordance with the Declaration of Helsinki, approved by the affiliated hospitals of the study, and followed the guidelines of the local institutional review board. The study received approval from the IRBs of the Athens University General Hospital “Attikon” (No. 280/17-5-21) and the University General Hospital of Patras (No. 6274/4-3-2021). Written informed consent was obtained from all participants involved in the study.

### 2.2 Subjects

Blood samples were prospectively collected from 193 seropositive patients across three Greek University Neurology Departments (First and Second Neurology Departments of the National and Kapodistrian University of Athens and the Neurology Department of the University of Patras). Serum samples were screened at Tzartos NeuroDiagnostics, Athens, using RIPA. Thirty disease-control sera (AChR-RIPA-negative) were also tested by L-CBA. For the present study, we recruited patients diagnosed with MG based on clinical manifestations of fluctuating muscle weakness and fatigue, along with the presence of antibodies against AChR (measured by RIPA; titer ≥ 1 nM), after excluding other possible differential diagnoses (8, 15). The MG Foundation of America (MGFA) Classification and the MGFA postintervention status were used to evaluate the maximum severity and outcome after treatment (16).

### 2.3 Antibody detection assays

All sera were initially tested by AChR-RIPA, following the manufacturer’s instructions (RSR-LTD, Cardiff, UK). RIPA-antibody values are expressed in nmol/L (nM); > 0.5 nmol/L is considered positive according to the manufacturer’s cut-off. However, for this study, only sera with titers ≥ 1 nM were included. F-CBA (Euroimmun, Lubeck, Germany) was performed according to the manufacturer’s instructions (serum dilution = 1:10).

For L-CBA (serum dilution = 1:10), HEK293 cells were transfected with all five human muscle AChR subunits and the intracellular anchoring protein rapsyn (6). Plasmids encoding the α-β-γ-δ-ϵ AChR subunits and rapsyn were transfected in a ratio of 2:1:1:1:1:1. Forty-eight hours posttransfection, cells were washed with Dulbecco’s modified Eagle’s medium (DMEM)/0.46% w/v *N*-(2-hydroxyethyl)-piperazine-*N*′-(2-ethanesulfonic acid) (HEPES) buffer (DMEM-HEPES) as described (6). CBA involved incubation of transfected cells with serum (1/10 dilution in 1% bovine serum albumin in DMEM-HEPES buffer). After 1 h, cells were washed three times with DMEM-HEPES buffer and fixed immediately with 4% paraformaldehyde (PFA) for 10 min. Fixed cells were incubated with rabbit anti-human IgG (H+L) (Thermo Fisher Scientific Inc., Waltham, MA, United States) at 1/750 dilution for 1 h, followed by incubation with goat anti-rabbit IgG Alexa Fluor-568 (Invitrogen) as the third antibody for 1 h (all at room temperature). CBA-negative sera were subsequently tested with separately transfected embryonic (α-β-γ-δ) and adult (α-β-ϵ-δ) AChRs. Microscopy analysis was performed under blinded conditions by three independent observers (KK, AGV, and JT or ES). The Olympus microscope CKX-41 was used, and images were analyzed using Infinity Analyze-6.5 Lumenera software. As negative controls, aquaporin 4 (AQP4)-transfected HEK293 cells were used. Samples from 21 sera in this study with RIPA titers of 1.0–2.8 nM were blindly retested by live and fixed CBA in a second laboratory at the University of Florence. These included six AChR-antibody-negative samples for both L- and F-CBA, seven F-CBA−/L-CBA+, and eight double-positive sera (L- and F-CBA), in addition to several negative controls. The in-house L-CBA sera were diluted 1:20, and L-CBA for the detection of antibodies against the fetal or adult AChR were tested separately. The sera were tested blindly by two independent observers (VD, FB).

Labeling of the secondary antibodies was scored as follows: (0) = no labeling; (1) = weak labeling of some transfected cells; (2) moderate labeling of more than 20% of transfected cells; and (3) strong labeling of approximately 50%–80% of transfected cells. Samples with a CBA score of ≥ 1 were considered positive. The Florence group used a similar scoring system (14).

### 2.4 Immunoadsorption assay with AChR-expressing cells

RIPA+/CBA− and RIPA+/CBA+ sera were preincubated for 3 h with live HEK293 cells expressing clustered AChR or AQP4 as a control on their surface, similar to the first step of the L-CBA procedure (i.e., transfected cells were attached to the well bottom). The cell supernatants were then tested for the presence of unbound AChR antibodies by RIPA. Sample volumes per cell-containing well were selected to ensure that a similar amount of anti-AChR antibodies (as determined by RIPA, as described above) was present, capable of precipitating 1,000–2,000 counts per minute (cpm) without preincubation with the cells. Comparison of the cpm from the supernatants of AChR-transfected cells with the cpm from the supernatants of control-transfected cells allowed calculation of the percentage of bound antibodies using the following formula: 100 × (1 − [{cpm of supernatant from AChR cells}/{cpm of supernatants from control cells}]) = percentage of AChR cell-bound antibodies.

### 2.5 Statistics

Statistical analysis and graphical presentation were performed using the following software packages: MedCalc ^®^ version 12.5 (MedCalc Software, Ostend, Belgium) and GraphPad Prism (GraphPad Software, San Diego, CA, USA). For comparison of paired samples, the paired *t*-test was used. All datasets are expressed as the mean ± standard deviation (SD), and *p*-values < 0.05 were considered statistically significant.

## 3 Results

### 3.1 Detection of antibodies against AChR clusters by CBA in RIPA-seropositive MG

The study included sera from 73 RIPA-positive MG patients with AChR antibodies, categorized into three groups based on RIPA levels: very low (1.0–1.7 nM, *n* = 25), low (2.0–2.8 nM, *n* = 11), and medium-to-high (3.0–4 nM, *n* = 37). After stratification based on RIPA results, the samples were retested using both L-CBA and F-CBA assays. Additionally, 120 sera with RIPA values > 4.0 nM but without clinical data were tested exclusively by L-CBA (**Supplementary Figure S1**). Furthermore, 30 disease-control RIPA-negative sera were tested by L-CBA, and all were found to be L-CBA negative. The sensitivity of the L-CBA for very low RIPA levels (1.0–1.7 nM) was found to be 80% (20 out of 25, 95% CI: 0.64, 0.96), while the sensitivity of the F-CBA was 40% (10 out of 25, 95% CI: 0.20, 0.60). These results indicate that L-CBA demonstrated satisfactory sensitivity in detecting very-low AChR antibody levels, whereas the sensitivity of F-CBA for sera with these RIPA titers was not satisfactory.

**Figure 1:**
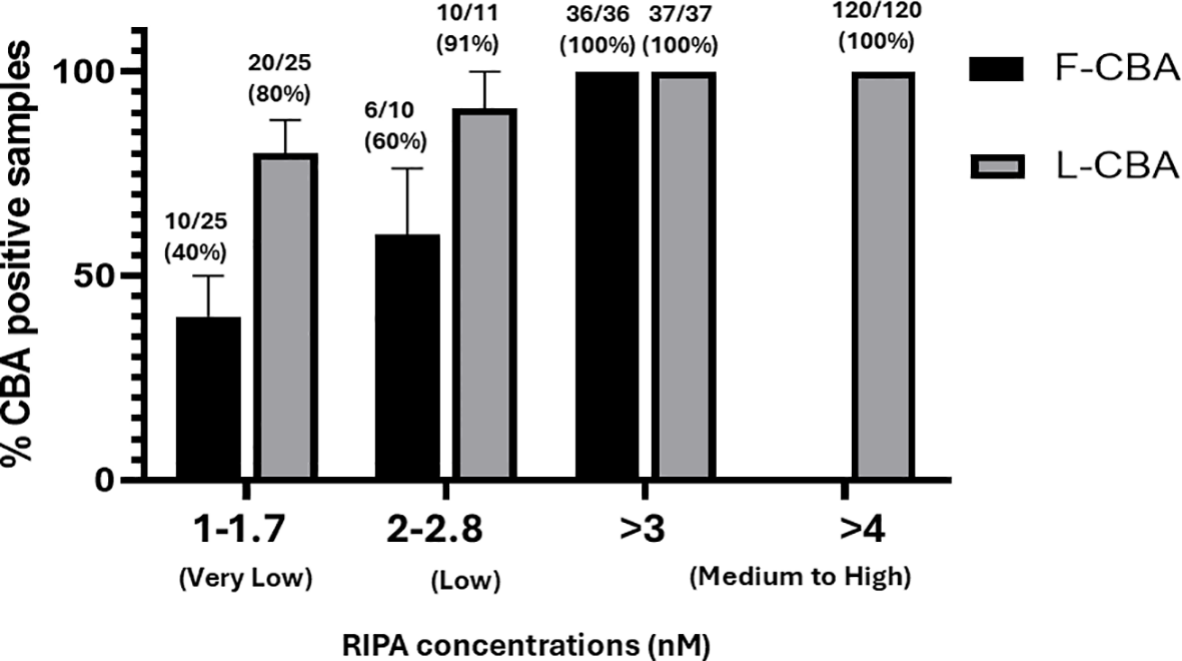
Detection of antibodies against AChR clusters by CBA in subgroups of RIPA-positive MG sera. Sera were classified into three subgroups based on their RIPA-determined values: very low, low, and medium-to-high. The corresponding RIPA values for each group are shown in the figure. F-CBA, fixed-cell CBA; L-CBA, live-cell CBA.

**Figure 2:**
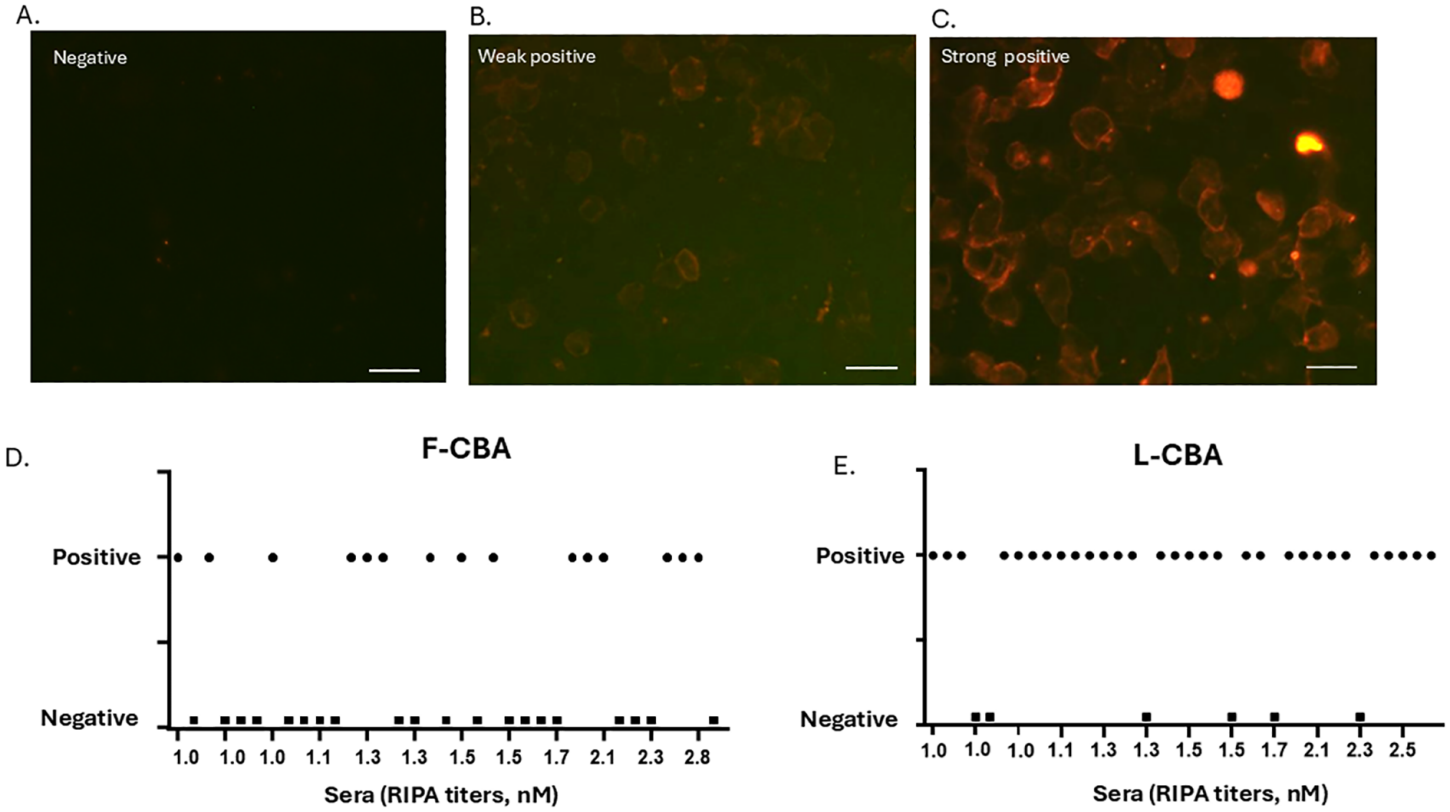
CBA staining patterns and detailed binding results of all individual sera. **(A–C)** Examples of negative, weak-positive, and strong-positive AChR antibody staining by L-CBA. HEK293 cells were transfected with the five human muscle AChR subunits and the intracellular anchoring protein rapsyn. Bound serum antibodies were visualized using a red-labeled secondary antibody. Live CBA shows a negative result (RIPA-positive/CBA-negative) **(A)**, a weak positive (weak labeling of several transfected cells) **(B)**, and a strong positive (strong labeling of several transfected cells) **(C)** result for cluster-AChR antibodies. Scale bar: 40 μm. **(D, E)** Plots showing individual CBA values (either L- or F-CBA) for the 36 very-low to low RIPA-seropositive MG patients included in the study. F-CBA, fixed-cell CBA; L-CBA, live-cell CBA.

In patients with low RIPA levels (2.0–2.8 nM), 10 out of 11 (91%, 95% CI: 0.59, 0.99) tested positive using the L-CBA, while six out of 10 (60%, 95% CI: 0.26, 0.87) tested positive using the F-CBA (**Figures 1**, **2**). All MG patients with AChR antibodies ≥ 3.0 nM by RIPA (*n* = 37) were positive with both CBAs (**Figure 1**). In addition, all 120 sera from patients with incomplete clinical data but with AChR antibody levels ≥ 4 nM by RIPA were tested by L-CBA and found to be positive (**Figure 1**).

In the Athens laboratory, there were no discrepancies between raters for the L-CBA assessment. For the F-CBA, initial discrepancies between raters were observed in four out of 71 cases. Of these, two were resolved after blinded reevaluation of the assay, while consensus was reached following retesting of the other two samples. Subsequently, weakly positive sera and all negative sera were blindly retested for both the Athens lab and the collaborating Florence laboratory (by VD and FB) for L- and F-CBA. The initial interlaboratory disagreement in three L-CBA and two F-CBA tests (out of 21 samples tested in both laboratories) was resolved after sample retesting.

**Figure 3:**
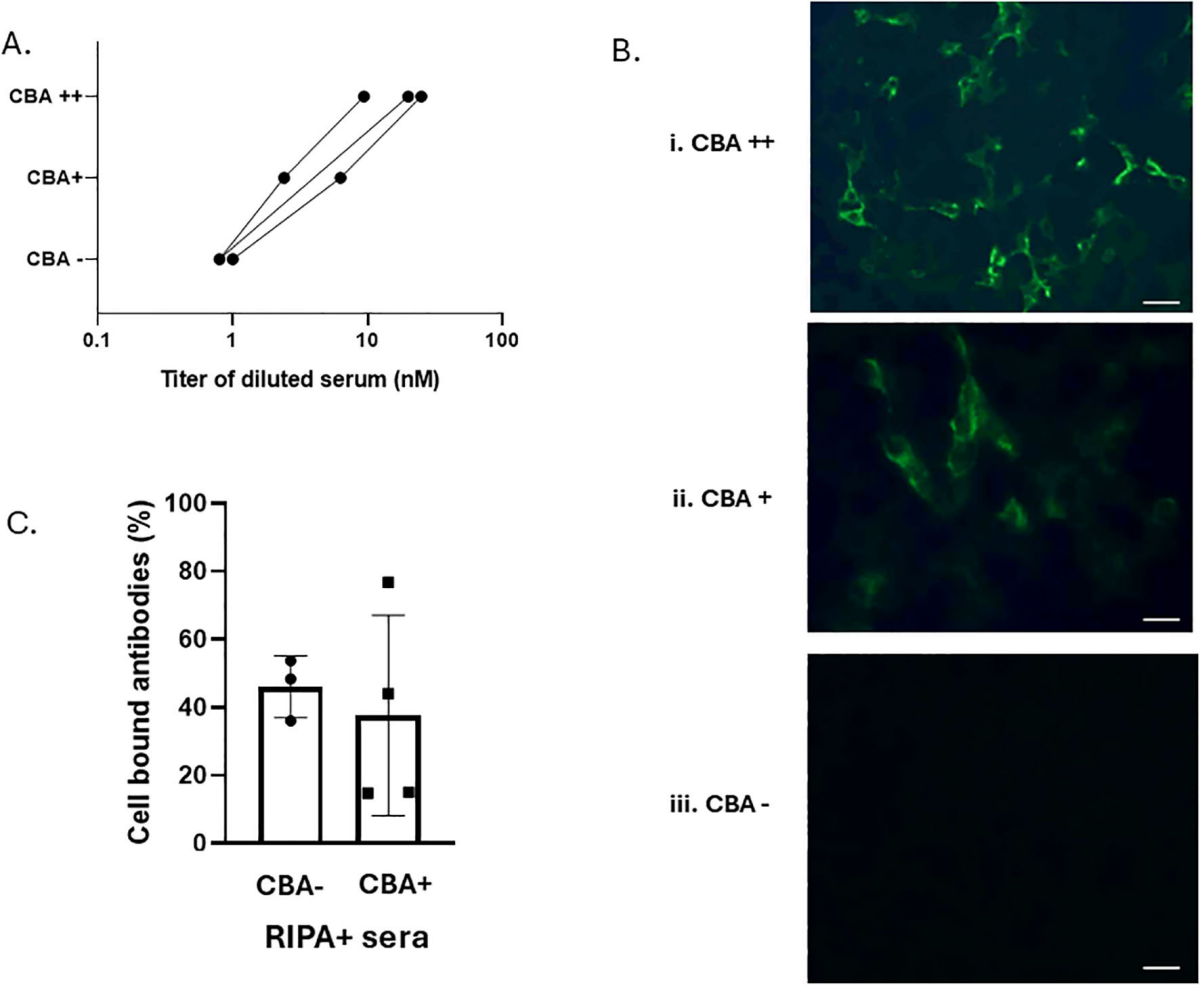
Experiments suggesting that AChR antibodies in low-titer RIPA+/CBA− sera are qualitatively similar to those in RIPA+/CBA+ sera. **(A)** Correlation of AChR-RIPA antibody titer and CBA binding for individual sera. Three high-titer sera were serially diluted to obtain moderate- and low-titer sera. Both undiluted and diluted sera were tested by cluster AChR F-CBA at a further 1:10 dilution, as used in all other experiments conducted at the Athens lab. **(B)** Examples of cell fluorescence staining in F-CBA++ (strongly positive), F-CBA+ (positive), and F-CBA− (negative) cases from **(A)**. Magnification: × 40. Scale bar: 40 μm. **(C)** Immunoadsorption on AChR clusters of AChR antibodies of CBA-negative and CBA-positive sera. Samples from three RIPA+/CBA− and four RIPA+/CBA+ sera were preincubated for 3 h with well-anchored cells expressing AChR clusters or the control protein AQP4, following the standard CBA protocol. The resulting cell supernatants were tested for unbound AChR antibodies using RIPA. Sample volumes per cell-containing well were adjusted to contain a similar amount of anti-AChR antibodies (as determined by RIPA), capable of precipitating 1,000–2,000 counts per minute (cpm) in the absence of preincubation. Comparison of the cpm values from the supernatants of AChR-transfected versus control-transfected cells yielded the percentage of bound antibodies (see Materials and methods; **Supplementary Table S1**). The average AChR-cell immunoadsorption was similar between the two groups: 46.0% ± 9.1% for the RIPA+/CBA− group and 42.7% ± 25.8% for the RIPA+/CBA+ group. No statistically significant difference was observed between the groups.

We selected three RIPA+/F-CBA+ sera with RIPA values ranging from 9 to 25 nM and performed serial dilutions to assess their performance in the F-CBA assay. As the serial dilutions were performed, the corresponding RIPA titers of the sera decreased. The diluted sera we then tested for F-CBA binding. **Figures 3A, B** show that while sera with RIPA titers ≥ 2.4 nM remained positive in the F-CBA assay, the same sera became negative when their titers approached 1.0 nM. These results suggest that some AChR antibodies detected at low concentrations by RIPA cannot be detected by the microscopy-based F-CBA (**Figures 3A, B**).

### 3.2 The AChR antibodies in low-titer RIPA+/CBA− sera bind to extracellular AChR epitopes, similar to those in RIPA+/CBA+ sera

We further conducted immunoadsorption assays to assess the presence of clustered AChR-binding antibodies in RIPA+/CBA− patients. Sera from three randomly selected low-titer RIPA+/CBA− patients and four medium-titer RIPA+/CBA+ patients were preincubated with cells expressing AChR clusters or the control protein AQP4. The culture supernatants were then tested for unbound AChR antibodies using RIPA, allowing calculation of the percentage of antibodies bound to the AChR-expressing cells. **Figure 3C** shows that AChR antibodies could be immunoadsorbed on intact AChR-expressing cells at comparable magnitude in both RIPA+/CBA− and RIPA+/CBA+ patient groups (average percentage of bound antibodies: 46.0% ± 9.1% for the CBA-negative sera [*n* = 3] versus 42.7% ± 25.8% for the CBA-positive sera, *n* = 4) (**Figure 3C**; **Supplementary Table S1**).

These data suggest that AChR antibodies detected at low concentrations by RIPA bind to extracellular epitopes in L-CBAs but are not detectable by microscopy-based L-CBA, possibly due to methodological limitations.

### 3.3 Clinical data of MG patients with AChR antibody: CBA-negative but RIPA-positive

All 73 MG patients were clinically diagnosed with MG. **Table 1** reports the clinical details of the MG patients who were positive for AChR antibodies by RIPA but negative by both L-CBA and F-CBA. These patients exhibited typical MG (50% ocular and 50% generalized MG). Of the patients, four out of six were men, and the mean age at disease onset was 65.16 years. At the time of sampling, all were undergoing immunotherapy (steroids or steroid-sparing agents). Most patients were stable during sample collection, including three patients with MGFA I, one with MGFA IIA, and one with MGFA IIB. Two patients underwent thymectomy, one of whom had a thymoma.

**Table 1:** Clinical features of patients who tested negative for both L-CBA and F-CBA*.

Study features	Patient Number	1^#^	2^#^	3	4	5	6
** AChR-Ab at study**	**RIPA (nM) at sample evaluation**	1.0	1.0	1.3	1.5	1.7	2.3
	**Immunotherapy at sample evaluation (Yes or No) including steroids**	Y	Y	Y	Y	Y	Y
	**RIPA (nM) at disease diagnosis**	126	11	1.3	2.3	2.5	2.3
**Demographic characteristics**	**Age at disease onset (Years)**	71	64	34	81	86	55
	**F:M**	M	M	F	M	M	F
**Clinical findings at any time**	**Diplopia (lateral gaze), R or L, (Yes or No)**	Y	N	Y	N	N	Y
	**Eyelid ptosis (Yes or No)**	Y	Y	Y	Y	Y	Y
	**Dysarthria (Yes or No)**	Y	N	N	Y	N	Y
	**Swallowing problem (Yes or No)**	Y	N	Y	N	N	Y
	**Respiratory muscle weakness/head drop/upper lower limbs**	N	N/N/N	N/N/Y	N	N	Y/Y/Y
	**Myasthenic crisis (Yes or No)**	N	N	N	N	N	N
	**Thymic involvement (hyperplasia/atrophy/thymoma)**	N	Atrophy	Hyperplasia	N	N	Thymoma (class B)
**Clinical findings at sample collection**	**Clinical classification (MGFA) at sample collection**	I	I	Asymptomatic	IIB	I	IIA
**Worse clinical findings**	**Highest clinical classification (MGFA)**	IIIb	I	IIIB	IIb	I	IIIB
**Electrophysiological observations (normal or pathological)**	**Repetitive nerve stimulation (Desmedt test)**	Not performed	N/A	(+)	Not performed	Not performed	(+)
	**Single fiber electromyography**	Not performed	N/A	N/A	Not performed	Not performed	N/A
**Treatment approaches and outcome**	**Response to pyridostigmine (remision, moderate response, for short time)**	No use	short time	short time	No use	No use	short time
	**Response to prednisolone (remission, moderate response)**	remission	moderate	moderate	remission	Moderate response	remission
	**Other Drugs (Other long-term therapy)**	Mycophenolate mofetil	none	Azathioprine/Mycophenolate mofetil	none	none	none
	**Short-term immunotherapy (PLEX / IVIG)**	N	N	N	N	N	Y (IVIG)
	**Thymectomy (Yes or No)**	N	N	Y	N	N	Y
	**Clinical evaluation at at last follow-up**	PR	I	Asymptomatic	PR	PR	PR

*PR, pharmacologic remission; MGFA, Myasthenia Gravis Foundation of America; PLEX, plasma exchange; IVIG, Intravenous Immunoglobulin; Y, Yes; N, No; N/A, non-applicable; AChR, acetylcholine receptor; RIPA, radioimmunoprecipitation assay; (+), positive examination; (-), negative examination; F-CBA, fixed-cell CBA; L-CBA, live-cell CBA; CBA; cell-based assay. ^#^MG patients with CBA-negative sera had also previous sera with higher RIPA titers (shown) which were found F-and L-CBA-positive.

## 4 Discussion

The present findings suggest that while F-CBA may not be sufficiently sensitive in detecting AChR antibodies in MG sera with low RIPA antibody titers, L-CBA appears to show higher sensitivity and more consistent results in identifying antibodies at low RIPA titers (1.0–2.8 nM). Of the patients with very-low to low RIPA titers (1.0–2.8 nM), 16.7% (six out of 36) were negative for L-CBA, while 54.3% (19 out of 35) were negative for F-CBA. Thus, RIPA and CBA can play complementary roles in the detection of AChR antibodies: RIPA is more reliable for detecting antibodies at low concentrations, while the clustered-AChR-CBA is more sensitive for identifying low-affinity antibodies in RIPA-negative samples (6).

Both assays have distinct features: RIPA is a quantitative test that detects AChR antibodies in solution, potentially identifying antibodies that bind to intracellular epitopes, whereas CBA detects only antibodies capable of binding to the cell surface, i.e., those that are potentially pathogenic. However, CBA is limited by its reliance on microscope-based techniques. Indeed, we observed a reduction of AChR antibody levels following serum immunoadsorption by AChR-bearing cells, with comparable magnitudes in both RIPA+/CBA− and RIPA+/CBA+ patients. This result suggests that the AChR antibodies of RIPA+/CBA− sera with low RIPA titers may also be pathogenic, rather than binding exclusively to intracellular epitopes. However, this does not exclude the possibility that some RIPA+/CBA− sera could bind only to intracellular epitopes, as suggested by Madisson et al. (17). While binding to cells expressing AChR indicates potential pathogenicity, it does not necessarily confirm CBA positivity, as concentration effects may influence the microscopy-based identification at lower dilutions.

The interpretation of CBA is limited by the visual grading method used in microscopy. In contrast, flow cytometry-based CBAs would offer the advantage of detecting low-concentration and low-affinity AChR antibodies, while providing quantitative data, and may prove to be more sensitive. Furthermore, modifications to the live and fixed CBA conditions could increase assay sensitivity without compromising the specificity. Such modifications could include, for example, the use of chaperons and/or AChR ligands, as seen in CBAs for neuronal AChRs (18), the use of alternative, more efficient secondary antibodies, or the use of smaller serum dilutions and/or larger incubation times, without increasing background staining.

Most reports on cluster AChR-CBA have studied RIPA-seronegative patients, while some recent reports have investigated the binding of RIPA-positive sera to AChR clusters by CBA, typically independent of RIPA titer. However, to our knowledge, the only previous paper addressing RIPA-AChR low-positive sera (13) reported that three out of 50 (6%) sera with RIPA AChR titers of 1.0–2.0 were negative at F-CBA. Collectively, we suggest that L-CBA should be preferred over F-CBA whenever possible, as cell fixation could distort the integrity of AChR, potentially affecting the binding of autoantibodies. While CBA, particularly with clustered AChR, offers enhanced sensitivity for detecting low-affinity antibodies, RIPA remains a crucial assay for detecting low antibody concentrations. The combination of both methods provides a more comprehensive and accurate diagnostic approach for MG.

In addition to RIPA and CBAs, commercial enzyme-linked immunosorbent assay (ELISA) for the detection of AChR antibodies is gaining ground and has been adopted by many diagnostic laboratories due to its relative ease of use. Several studies have demonstrated the considerable validity of commercial ELISAs; however, their overall sensitivity and specificity are inferior to those of RIPA and CBAs (19, 20). Therefore, we suggest that RIPA or CBAs should be preferred or used to confirm the ELISA result, particularly when the ELISA result does not align with the clinical phenotype.

One limitation of our study is that we did not include patients with low RIPA titer AChR at disease onset, nor did we include a control group. Additionally, the absence of neurophysiological tests to support the diagnosis of MG in some patients with RIPA+/CBA− AChR antibodies represents a significant limitation.

Overall, we conclude that both AChR antibody assays (RIPA and CBA), if available in a diagnostic laboratory, should ideally be part of the routine diagnosis of MG. We propose that if RIPA is the first choice and the results are positive, correlating well with the clinical phenotype, then CBA may not be required. However, if a positive RIPA result is inconsistent with the clinical presentation, CBA should be performed. It is important to consider that a low RIPA titer may lead to negative F-CBA results due to issues with antibody concentration, rather than due to the pathogenicity of the antibodies. In such cases, the use of L-CBA would be necessary for the final decision. However, a limited number of samples might also be low-titer AChR-RIPA-positive and L-CBA-negative due to either nonpathogenic (potentially cytoplasmic) antibodies or insufficient concentrations of pathogenic extracellular antibodies for a positive CBA signal. Alternatively, if CBA is the first choice, a positive CBA result would make RIPA useful only for titration and potentially for disease monitoring. In the case of negative CBA results, in addition to testing for MuSK and LRP4 antibodies, AChR-RIPA should also be considered.

## Data Availability

The original contributions presented in the study are included in the article/**Supplementary Material**. Further inquiries can be directed to the corresponding authors.

## Supplementary material

Supplementary Figure 1 Schematic representation of the selection process of all serum samples included in the present study.
