# Supplementary figures and images for: Efficiency of cell-based assays in detecting AChR antibodies in myasthenia gravis sera with low antibody concentrations as determined by radioimmunoprecipitation assay
Source: Front Immunol. 2025 May 28;16:1459423. doi: 10.3389/fimmu.2025.1459423 (PMC12151784; doi:10.3389/fimmu.2025.1459423)

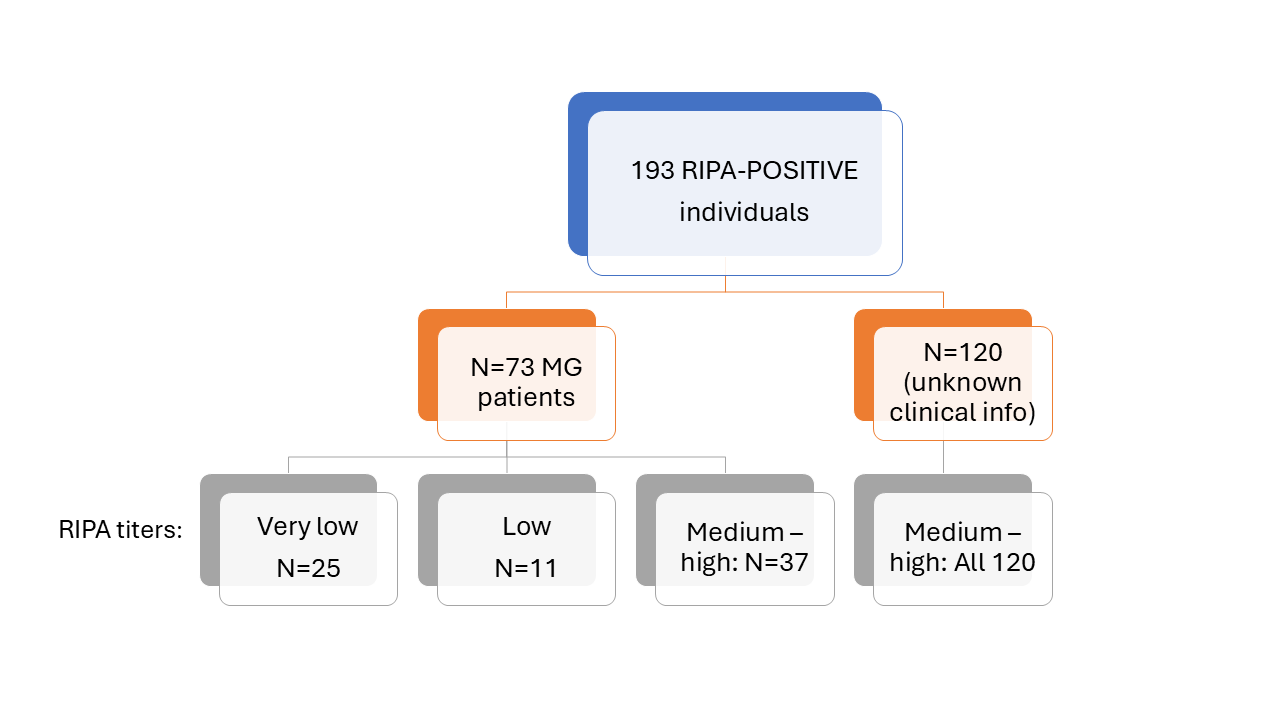

Supplement: Supplementary Figure 1 — Schematic representation of the selection process of all serum samples included in the present study. [file Image1.tif]
