# Supplementary material for: Efficiency of cell-based assays in detecting AChR antibodies in myasthenia gravis sera with low antibody concentrations as determined by radioimmunoprecipitation assay
Source: Front Immunol. 2025 May 28;16:1459423. doi: 10.3389/fimmu.2025.1459423 (PMC12151784; doi:10.3389/fimmu.2025.1459423)
Supplement: Supplementary file 2 [file Table1.docx]

**Supplementary Table 1**

Immunoadsorption of AChR antibodies of CBA-negative and CBA-positive sera on AChR-cluster expressing cells. Table shows Δcpm measured by RIPA of sera preadsorbed on control (AQP4) or AChR expressing cells.

| **Patients** | **Average RIPA-precipitated Δcpm*** | | | **Fraction of non-adsorbed Abs** | **Adsorbed antibodies (%)** |
| --- | --- | --- | --- | --- | --- |
|  | **Untreated serum** | **Supernatant of AQP4 expressing control-cells** | **Supernatant of AChR expressing cells** |  |  |
| **CBA- patients** |  |  |  |  |  |
| Patient 1 | 985 | 971 | 501 | 0,52 | 48,4 |
| Patient 2 |  | 1271 | 589 | 0,46 | 53,7 |
| Patient 3 |  | 1041 | 666 | 0,64 | 36,0 |
| Average |  |  |  | 0,54 | **46,0** |
| SD |  |  |  | 0,09 | **9,1** |
|  |  |  |  |  |  |
| **CBA+ patients** |  |  |  |  |  |
| Patient 4 | 1012 | 988 | 840 | 0,85 | 15,0 |
| Patient 5 |  | 1119 | 730 | 0,65 | 34,8 |
| Patient 6 |  | 2102 | 1176 | 0,56 | 44,0 |
| Patient 7 |  | 1540 | 357 | 0,23 | 76,8 |
| Average |  |  |  | 0,57 | **42,7** |
| SD |  |  |  | 0,26 | **25,8** |

*Average Δcpm of the control-cell (AQP4-treansfected cells) treated and AChR-cell (AChR-transfected cells) treated sera (see Methods section).

SD: standard deviation, cpm: counts per minute, Δcpm: AChR specific cpm, i.e. cpm precipitated by patient serum, minus the average cpm precipitated by four control healthy individuals’ sera (330 cpm).
